# Detrimental relevance of *Helicobacter pylori* infection with sarcopenia

**DOI:** 10.1186/s13099-021-00464-y

**Published:** 2021-11-15

**Authors:** Shou-En Wu, Wei-Liang Chen

**Affiliations:** 1 Department of Dermatology, Tri-Service General Hospital; and School of Medicine, National Defense Medical Center, Taipei, Taiwan, Republic of China; 2 Division of Family Medicine, Department of Family and Community Medicine, Tri-Service General Hospital; and School of Medicine, National Defense Medical Center, Taipei, Taiwan, Republic of China; 3 Division of Geriatric Medicine, Department of Family and Community Medicine, Tri-Service General Hospital; and School of Medicine, National Defense Medical Center, Number 325, Section 2, Chang-gong Rd, Nei-Hu District, Taipei, 114 Taiwan, Republic of China; 4 Department of Biochemistry, National Defense Medical Center, Taipei, Taiwan, Republic of China

**Keywords:** *Helicobacter pylori*, Sarcopenia, Muscle mass, Eradication therapy

## Abstract

**Background:**

*Helicobacter pylori* (*H. pylori*), Gram negative microaerophilic bacteria, is a well-known pathogen of many gastrointestinal diseases. But several emerging evidences suggest it role in numerous other extra-gastric diseases. The current study investigates the relationship between *H. pylori* infection and sarcopenia, a clinical condition characterized by the loss of mass and function of skeletal muscle. A total of 3453 eligible participants from the Third National Health and Nutrition Examination Survey (NHANES III), the United States, were enrolled. Based on the serum laboratory results, subjects were categorized into three groups: normal (without evidence of any *H. pylori* infection), anti-*H. pylori* IgG positive [*H. pylori* (+)], and concurrent anti-*H. pylori* IgG and anti-cytotoxin-associated gene A IgG positive [CagA (+)]. Sarcopenia was determined as having a skeletal muscle index (SMI) value that is more than 1 standard deviation away from the mean value of sex-specific, healthy young adults between 20 and 39 years old. Risk of sarcopenia and its components are compared between subgroups.

**Results:**

Odds ratios (OR) for confirmed diagnosis of sarcopenia were higher in *H. pylori* (+) (OR = 2.052, 95% CI 1.697–2.481, p < 0.001) and CagA (+) (OR = 1.585, 95% CI 1.278–1.965, p < 0.001) groups. Moreover, negative beta regression coefficient of SMI were shown in *H. pylori* (+) (β: − 0.023, p < 0.001) and CagA (+) (β: − 0.017, p < 0.001). Sub-analyses which categorized participants by gender revealed that absolute value of beta regression coefficient for SMI were higher in female in *H. pylori* (+) subgroup (β: − 1.745 in male and − 2.942 in female, p were both < 0.001), and the CagA (+) subgroup (β: − 1.407 in male and − 2.159 in female, p were both < 0.001).

**Conclusions:**

Positive serum *H. pylori* infectious markers including anti-*H. pylori* antibody and CagA seropositivity are correlated with sarcopenia and low muscle quantity. Therefore, *H. pylori* eradication therapy may bring benefits to sarcopenia patients with concurrent active *H. pylori* infection.

## Background

*Helicobacter pylori* (*H. pylori*) is a Gram-negative, helical-shaped bacterium that colonizes over 50% of the world’s population [1]. Nevertheless, only 10–20% infected patients develop peptic ulcer disease, and 1–2% develop gastric malignancy [2, 3]. Aside from being a renowned pathogen of gastrointestinal diseases, there is growing evidence of *H. pylori*’s role in various extra-gastric diseases [4]. Literature has reported its relationship with neurological disorder (e.g., Alzheimer's diseases and multiple sclerosis) [5], dermatological diseases (e.g., rosacea) [6], hematologic diseases (e.g., iron deficiency anemia and primary immune thrombocytopenia) [7, 8], and so on. Manifestations in numerous sites of the body illustrate that *H. pylori* may induce not only local but systemic impacts that involve complex interactions [9].

Recently, societal aging has raised much health concern, including the increasing prevalence of sarcopenia, a muscle-related disorder that raises severe public health concern such as disability, falls, hospitalization, and mortality elderly population [10, 11]. This arouses our interest of whether muscle, an extra-gastric organ, also possesses an association with *H. pylori*. A little research has been focused on the effect of *H. pylori* infection on muscle physiology [12], and no further evidence verifies the idea. Examining the association within may not only validate that *H. pylori* infection triggers systemic health effects, but also provides an extended understanding of the pathogenesis of sarcopenia that could possibly facilitate an alternative treatment strategy. The aim of the present study is exploring the relationship between serum *H. pylori* infectious markers and sarcopenia.

## Results

### Demographic information of the study population

In Table 1, participants were classified into three groups according to blood test results for *H. pylori*, namely normal, anti-*H. pylori* IgG positive, and concurrent anti-*H. pylori* and anti-cytotoxin-associated gene A (CagA) IgG positive. Most parameters including gait speed (time used for 8-feet walk), body mass index (BMI), C-reactive protein (CRP) levels or fasting glucose levels showed no statistical difference among groups. Few that showed difference were skeletal muscle index (SMI), race, diabetes mellitus, and sarcopenia. SMI were the lowest in the CagA (+) group(41.82 ± 10.98), and the highest in normal group (44.69 ± 9.83,p value < 0.001). The proportion of non-Hispanic black and Mexican–American were higher in the CagA (+) (non-Hispanic black:29.9%, Mexican–American:22.4%) and *H. pylori* (+) (non-Hispanic black:22.0%, Mexican–American:20.6%) groups compared with the normal group (non-Hispanic black:16.6%, Mexican–American:18.4%) (p < 0.001). The highest proportion of participants having diabetes mellitus appeared in the CagA (+) group (17.2%). Larger proportion of participants were diagnosed with sarcopenia in *H. pylori* (+) (10.9%) and CagA (+) (10.4%) groups comparing to the normal group (4.5%) (p < 0.001).

**Table 1:** Characteristics of study participants in normal, *H. pylori* (+), and CagA (+) groups

	Normal^‡^ (n = 2805)	*H. pylori* (+)^§^ (n = 514)	CagA (+)^¶^ (n = 134)	P value
Continuous values†				
Age (years)	71.37 ± 7.99	71.74 ± 8.07	71.43 ± 6.97	0.770
BMI (kg/m^2^)	27.24 ± 5.10	26.72 ± 4.66	27.22 ± 5.13	0.127
SMI (kg/m^2^)	44.69 ± 9.83	42.82 ± 10.97	41.82 ± 10.98	< 0.001
Time used for 8-feet walk(s)	3.94 ± 2.34	3.80 ± 1.48	3.86 ± 1.61	0.542
Plasma CRP levels	0.59 ± 1.07	0.51 ± 0.73	0.51 ± 0.68	0.156
Plasma glucose levels	110.54 ± 43.88	107.61 ± 44.24	112.02 ± 51.24	0.464
Categorical values^†^				
Male	52.7%	46.4%	49.1%	0.254
Race				
Non-Hispanic white	61.8%	55.5%	44.8%	< 0.001
Non-Hispanic black	16.6%	22.0%	29.9%	
Mexican–American	18.4%	20.6%	22.4%	
Other	3.2%	1.8%	2.9%	
Low muscle strength (difficulty lifting 10 pounds)	29.8%	33.7%	31.3%	0.883
Smoker	16.4%	18.8%	15.2%	0.127
Doctor ever told to have arthritis	44.5%	42.9%	43%	0.704
Doctor ever told to have asthma	6.3%	6.7%	5.6%	0.069
Diabetes mellitus	14.3%	15.3%	17.2%	0.049
Sarcopenia	126 (4.5%)	56 (10.9%)	14 (10.4%)	< 0.001

^†^Continuous values were expressed as mean and standard deviation; Categorical values in the categorical variables were expressed in number and percentage (%)

^‡^Normal: without evidence of positive anti-*H. pylori* IgG or anti-CagA IgG

^§^*H. pylori* (+): positive anti-*H. pylori* IgG in serum samples

^¶^CagA (+): concurrent positive anti-cytotoxin-associated gene A IgG and positive anti-*H. pylori* IgG in serum samples

BMI: Body mass index; SMI: Skeletal muscle index; CRP: C-reactive protein

### Logistic regression models of association between serum anti-*H. pylori* IgG status, anti-CagA IgG status and sarcopenia

To examine the association between serum *H. pylori* markers and sarcopenia, logistic regression models are used to analyze the categorical variables, including confirmed diagnosis of sarcopenia (defined by low muscle mass in the present study), low gait speed and low muscle strength (Table 2). Odds ratios (OR) for confirmed diagnosis of sarcopenia were higher in *H. pylori* (+) (OR 2.052, 95% CI 1.697–2.481, p < 0.001) and CagA (+) (OR = 1.585, 95% CI 1.278–1.965, p < 0.001) groups. ORs of low gait speed and low muscle strength didn’t reveal statistical significance. The above results illustrated the higher probability of sarcopenia in H. pylori (+) and CagA (+) groups. The non-significant results in gait speed and muscle strength imply that muscle mass may be the first or decisive factor in certain scenarios.

**Table 2:** Odds ratios (OR) for sarcopenia and its components in normal, *H. pylori* (+), and CagA (+) groups

Normal^‡^	Model 1^†^	Model 2	Model 3	Model 4
	Ref	Ref	Ref	Ref
Sarcopenia				
*H. pylori* (+)^§^				
OR	1.832 (1.578,2.128)	1.860 (1.577,2.194)	2.035 (1.685,2.459)	2.052 (1.697,2.481)
P value	< 0.001	< 0.001	< 0.001	< 0.001
CagA (+)^¶^				
OR P value	1.389 (1.174,1.643)	1.433 (1.189,1.727)	1.563 (1.261,1.936)	1.585 (1.278,1.965)
P value	< 0.001	< 0.001	< 0.001	< 0.001
Low gait speed (< 0.8 m/s)				
*H. pylori* (+)				
OR	1.001 (0.862,1.152)	1.002 (0.862,1.165)	0.975 (0.838,1.135)	0.978 (0.840,1.138)
P value	0.966	0.980	0.748	0.774
CagA (+)				
OR	1.014 (0.859,1.198)	1.099 (0.924,1.307)	1.082 (0.908,1.288)	1.082 (0.908,1.289)
P value	0.869	0.288	0.379	0.378
Difficulty lifting 10 pounds				
*H. pylori* (+)				
OR	1.195 (0.927,1.540)	1.184 0.917,1.527)	1.189 (0.921,1.536)	1.184 (0.916,1.530)
P value	0.170	0.195	0.184	0.197
CagA (+)				
OR	1.021 (0.756,1.379)	1.012 (0.746,1.373)	1.010 (0.744,1.370)	0.998 (0.735,1.355)
P value	0.890	0.938	0.949	0.990

^†^Model 1 = unadjusted

Model 2 = Adjusted for age, gender, race

Model 3 = Adjusted for age, gender, race, BMI, serum C-reactive protein, serum glucose

Model 4 = Adjusted for age, gender, race, BMI, serum C-reactive protein, serum glucose, smoking, arthritis, asthma, diabetes mellitus

^‡^Normal: without evidence of positive anti-*H. pylori* IgG or anti-CagA IgG

^§^*H. pylori* (+): positive anti-*H. pylori* IgG in serum samples

^¶^CagA (+): concurrent positive anti-cytotoxin-associated gene A IgG and positive anti-*H. pylori* IgG in serum samples

### Linear regression models of association between serum anti-*H. pylori* IgG status, anti-CagA IgG status and sarcopenia

Next, we examined the association between serum *H. pylori* markers and sarcopenia by calculating muscle mass, muscle strength and gait speed as continuous variables (Table 3). Linear regression models are used in current analysis. Beta regression coefficient of SMI in *H. pylori* (+) (β: − 0.023, p < 0.001) and CagA (+) (β: − 0.017, p < 0.001) were of negative values. This explains the negative association between *H. pylori* infection and muscle mass, i.e., lower muscle mass in these two groups. On the other hand, results of gait speed did not reveal a similar trend with p values not reaching the statistical significance.

**Table 3:** Association between components of sarcopenia and *H. pylori* markers in normal, *H. pylori* (+), and CagA (+) groups

Normal^‡^	Model 1^†^	Model 2	Model 3	Model 4
	Ref	Ref	Ref	Ref
Skeletal muscle index (kg/m^2^)				
*H. pylori* (+)^§^				
β coefficient	− 0.015 (− 0.022, − 0.009)	− 0.021 (− 0.027, − 0.016)	− 0.023 (− 0.028, − 0.018)	− 0.023 (− 0.029, − 0.018)
P value	< 0.001	< 0.001	< 0.001	< 0.001
CagA (+)^¶^				
β coefficient	− 0.014 (− 0.022, − 0.007)	− 0.016 (− 0.022, − 0.009)	− 0.017 (− 0.023, − 0.011)	− 0.017 (− 0.023, − 0.011)
P value	< 0.001	< 0.001	< 0.001	< 0.001
Gait speed (m/s)				
*H. pylori* (+)				
β coefficient	0.019 (− 0.006, 0.043)	0.013 (− 0.010, 0.036)	0.009 (− 0.013, 0.032)	0.010 (− 0.013, 0.033)
P value	0.132	0.266	0.417	0.385
CagA (+)				
β coefficient	0.014 (− 0.030, 0.058)	0.029 (− 0.013, 0.071)	0.027 (− 0.015, 0.069)	0.027 (− 0.015, 0.069)
P value	0.536	0.181	0.209	0.206

^†^ Model 1 = unadjusted

Model 2 = Adjusted for age, gender, race

Model 3 = Adjusted for age, gender, race, BMI, serum C-reactive protein, serum glucose

Model 4 = Adjusted for age, gender, race, BMI, serum C-reactive protein, serum glucose, smoking, arthritis, asthma, diabetes mellitus

^‡^Normal: without evidence of positive anti-*H. pylori* IgG or anti-CagA IgG

^§^*H. pylori* (+): positive anti-*H. pylori* IgG in serum samples

^¶^CagA (+): concurrent positive anti-cytotoxin-associated gene A IgG and positive anti-*H. pylori* IgG in serum samples

### Anti-*H. pylori* IgG status, anti-CagA IgG status and frequency of sarcopenia among males and females

To examine whether gender difference exists in such relationship, subgroup analysis categorizing the participants by gender is performed (Tables 4 and 5). ORs of confirmed diagnosis of sarcopenia were similar in males and females in both *H. pylori* (+) (OR = 2.164 in male and 2.137 in female, p values were both < 0.001) and CagA (+) (OR = 1.782 in male and 1.650 in female, p values were = 0.010 and 0.012, respectively). ORs of low gait speed and low muscle strength (difficulty lifting 10 pounds) didn’t reveal statistical significance. As for SMI, absolute value of beta regression coefficient were higher in female in *H. pylori* (+) subgroup (β: − 1.745 in male and − 2.942 in female, p were both < 0.001), and the CagA (+) subgroup (β: − 1.407 in male and − 2.159 in female, p were both < 0.001).

**Table 4:** Odds ratios (OR) for sarcopenia and its components in normal, *H. pylori* (+), and CagA (+) groups categorized by gender

	Male (n = 1765)			Female (n = 1688)		
	Normal (n = 1455)	*H. pylori* (+) (n = 233)	CagA (+) (n = 77)	Normal (n = 1350)	*H. pylori* (+) (n = 281)	CagA (+) (n = 57)
Sarcopenia						
Model 1						
OR	Ref	2.132 (1.441, 3.153)	1.801 (1.171, 2.770)	Ref	2.127 (1.508, 3.002)	1.678 (1.143, 2.463)
P value	Ref	< 0.001	0.007	Ref	< 0.001	0.008
Model 2						
OR	Ref	2.085 (1.408, 3.087)	1.745 (1.130, 2.695)	Ref	2.119 (1.501, 2.992)	1.652 (1.124, 2.430)
P value	Ref	< 0.001	0.012	Ref	< 0.001	0.011
Model 3						
OR	Ref	2.161 (1.456,3.208)	1.776 (1.148,2.748)	Ref	2.155 (1.525,3.047)	1.679 (1.141,2.470)
P value	Ref	< 0.001	0.010	Ref	< 0.001	0.009
Model 4						
OR	Ref	2.164 (1.457, 3.214)	1.782 (1.151, 2.759)	Ref	2.137 (1.509, 3.026)	1.650 (1.118, 2.436)
P value	Ref	< 0.001	0.010	Ref	< 0.001	0.012
Low gait speed (< 0.8 m/s)						
Model 1						
OR	Ref	1.137 (0.843, 1.534)	0.923 (0.571, 1.493)	Ref	1.291 (0.991, 1.682)	1.597 (0.909, 2.807)
P value	Ref	0.400	0.744	Ref	0.058	0.103
Model 2						
OR	Ref	1.197 (0.877, 1.633)	0.794 (0.482, 1.308)	Ref	1.301 (0.990, .710)	1.509 (0.847, 2.690)
P value	Ref	0.256	0.365	Ref	0.059	0.163
Model 3						
OR	Ref	1.288 (0.940, 1.765)	0.794 (0.480, 1.313)	Ref	1.335 (1.014, 1.757)	1.552 (0.838, 2.773)
P value	Ref	0.115	0.368	Ref	0.039	0.138
Model 4						
OR	Ref	1.287 (0.938, 1.765)	0.800 (0.483, 1.326)	Ref	1.336 (1.014, 1.759)	1.558 (0.872, 2.783)
P value	Ref	0.118	0.388	Ref	0.039	0.135
Low muscle strength (difficulty lifting 10 pounds)						
Model 1						
OR	Ref	1.246 (0.761, 2.042)	0.787 (0.308, 2.012)	Ref	1.373 (0.867, 2.174)	1.264 (0.519, 3.080)
P value	Ref	0.382	0.617	Ref	0.176	0.606
Model 2						
OR	Ref	1.241 (0.757, 2.034)	0.796 (0.311, 2.039)	Ref	1.382 (0.872, 2.191)	1.240 (0.506, 3.038)
P value	Ref	0.392	0.635	Ref	0.169	0.638
Model 3						
OR	Ref	1.250 (0.761, 2.051)	0.782 (0.304, 2.013)	Ref	1.385 (0.872, 2.201)	1.227 (0.500, 3.009)
P value	Ref	0.378	0.610	Ref	0.167	0.655
Model 4						
OR	Ref	1.279 (0.776, 2.108)	0.791 (0.306, 2.044)	Ref	1.344 (0.844, 2.140)	1.153 (0.467, 2.850)
P value	Ref	0.334	0.629	Ref	0.213	0.757

^†^Model 1 = unadjusted

Model 2 = Adjusted for age, race

Model 3 = Adjusted for age, race, BMI, serum C-reactive protein, serum glucose

Model 4 = Adjusted for age, race, BMI, serum C-reactive protein, serum glucose, smoking, arthritis, asthma, diabetes mellitus

**Table 5:** Association between SMI and *H. pylori* markers in normal, *H. pylori* (+), and CagA (+) groups categorized by gender

	Male (n = 1765)			Female (n = 1661)		
	Normal (n = 1455)	*H. pylori* (+) (n = 233)	CagA (+) (n = 77)	Normal (n = 1323)	*H. pylori* (+) (n = 281)	CagA (+) (n = 57)
Skeletal muscle index(SMI)						
Model 1						
β coefficient	Ref	− 1.525 (− 2.392, − 0.658)	− 1.563 (− 2.546, − 0.581)	Ref	− 2.369 (− 3.323, − 1.414)	− 1.476 (− 2.591, − 0.361)
P value	Ref	0.001	0.002	Ref	< 0.001	0.010
Model 2						
β coefficient	Ref	− 1.266 (− 2.101, − 0.432)	− 1.321 (− 2.271, − 0.371)	Ref	− 2.601 (− 3.539, − 1.663)	− 1.676 (− 2.776, − 0.576)
P value	Ref	0.003	0.006	Ref	< 0.001	0.003
Model 3						
β coefficient	Ref	− 1.733 (− 2.406, − 1.060)	− 1.390 (− 2.158, − 0.622)	Ref	− 2.926 (− 3.747, − 2.106)	− 2.144 (− 3.107, − 1.181)
P value	Ref	< 0.001	< 0.001	Ref	< 0.001	< 0.001
Model 4						
β coefficient	Ref	− 1.740 (− 2.413, − 1.067)	− 1.407 (− 2.175, − 0.640)	Ref	− 2.942 (− 3.763, − 2.122)	− 2.159 (− 3.123, − 1.194)
P value	Ref	< 0.001	< 0.001	Ref	< 0.001	< 0.001

^†^Model 1 = unadjusted

Model 2 = Adjusted for age, race

Model 3 = Adjusted for age, race, BMI, serum C-reactive protein, serum glucose

Model 4 = Adjusted for age, race, BMI, serum C-reactive protein, serum glucose, smoking, arthritis, asthma, diabetes mellitus

## Discussion

The primary finding of this study is the positive association between sarcopenia and two *H. pylori* infection markers, serum anti-*H. pylori* IgG status and CagA seropositivity. Participants with positive anti-*H. pylori* IgG status and anti-CagA IgG status have higher risk of developing sarcopenia and low muscle mass (determined by SMI), whereas the other two indications of sarcopenia (i.e., low muscle strength and low physical performance) didn't show significant relationship. Previous research has not put much attention on the association between *H. pylori* infection and sarcopenia. The merely one that we came across was a study performed in Seoul, Korea which revealed elderly women receiving *H. pylori* eradication therapy had decreased risk of low skeletal muscle mass [12]. However, it was limited to female and did not further evaluate the status of muscle function. To the best of our knowledge, we are the first to propose the link between *H. pylori* infection and sarcopenia.

There are three essential findings in our study that are worth discussing. First, we demonstrated that not only anti-*H. pylori* IgG status but also CagA seropositivity relates to sarcopenia. Anti-CagA IgG status may not be considered as a marker for infection as *cagA*-negative strains also exist. However, CagA is one of the virulence factors that strongly links to more severe conditions like peptic ulcer and gastric cancer [13]. The CagA protein is injected into the host cell by a type 4 secretion system. Inside the cell, the protein activates a cascade of downstream events resulting in induce altered cell morphology, cell proliferation and damaged cell junction. Clinicians may consider shortening the screening intervals for sarcopenia in patients who are concurrent seropositive for anti-*H. pylori* and anti-CagA (+) antibodies. Second, low muscle mass is the only component of sarcopenia that showed correlation with *H. pylori* infection. This may be due to the direct influence of *H. pylori* infection on muscle quality and quantity (possible mechanisms are described in the following section). Nevertheless, if we conduct a longitudinal study that tracks patients over a period of time, the impact on muscle function may appear. Third, the absolute value of beta coefficient was higher in female in *H. pylori* (+) and CagA (+) subgroup, illustrating the greater decrease in SMI in female patients of *H. pylori* infection. This is quite interesting finding as several previous studies mostly revealed that *H. pylori* infected males have higher risk of developing gastric-related diseases including chronic gastritis and gastric cancer [14, 15]. Our finding implied there may be a different trend of gender predominance in sarcopenia, which is a muscle disease primarily found in elderly population. Estrogen may be the key character here. Previous literature has illustrated the protective role of estrogen against *H. pylori* infection [16, 17], but the average age of our participants (71.37 ± 7.99 years old), were old enough to be in postmenopausal stage, who have lost the privilege of hormone protection. This might lead to a more vulnerable status in elderly female, which turned out to have a higher degree of muscle decline after *H. pylori* infection.

Possible mechanisms underlying the interrelation are as follows. First, *H. pylori* infection results in dysbiosis of gut microbiome, and these alterations may further cause sarcopenia through a gut-muscle axis that was mentioned in previous articles [18–20]. Microbiota in the gastric system maintains a delicate balance in immunity and health by producing multiple mediators that influences not only the gastro-intestinal tract but distal organs. Muscle, inevitably, is one of the victims if dysregulation occurs. Secondly, *H. pylori* induces fluctuations of hormones that regulates energy homeostasis including ghrelin and leptin [21–23]. Ghrelin, mainly produced by gastric endocrine cells, stimulates appetite and the release of growth hormone. Evidence showed decreased ghrelin levels in sarcopenia patients [24], while another study proved improvement of physical decline through ghrelin administration [25]. On the other hand, leptin is the product of adipocytes and gastric endocrine cells, and modulates fat storage and appetite control. Studies have discussed changes of leptin levels in sarcopenia patients, and recognized its role in aging muscle [26]. Collectively, ghrelin and leptin may bridge *H. pylori* and sarcopenia altogether. Third, *H. pylori* infection leads to inflammatory response through cytokines including interleukin-1,6,12 (IL-1, IL-6, IL-12), tumor necrosis factor-α(TNF-α), and interferon-γ (IFN-γ) [27]. The initial local inflammation could spread as these cytokines circulates in the bloodstream. Chronic inflammatory condition brings negative effects to muscle mass and strength, ultimately giving rise to the occurrence of sarcopenia [28]. Last but not least, *H. pylori* infection decreases absorption of vital nutrients, which in turn causes growth retard in childhood, and unsurprisingly, sarcopenia in the elderly [29]. The above speculations are yet to be verified, but fairly reasonable to explain the association between *H. pylori* infection and sarcopenia.

The importance of certain investigation is the growing evidence of extra-gastric diseases caused by *H. pylori* [4]. Aside from the well-known determinant pathogen of chronic gastritis, peptic ulcer, gastric adenocarcinoma, and gastric mucosa associated lymphoid tissue (MALT) lymphoma [30, 31], recent studies imply that *H. pylori* may not just pose threat to the body locally but rather have a great impact systemically. As a potential influencing factor in neurological, dermatological, and hematologic diseases [5, 7, 8, 32], the mechanism of *H. pylori* pathogenicity is apparently multifactorial and complicated. Fortunately, current guidelines of treatment regimens can achieve almost 90% eradication rate [33]. In other words, the prognosis of diseases that were discovered to have association with *H. pylori* infection is promising. The target of our study, sarcopenia, is a disorder that raises public health concern such as disability, falls, hospitalization, and mortality [10, 11], but still awaits effective therapeutic strategies due to multifactorial etiology. According to the findings of our study, we propose that anti-*H. pylori* IgG seropositive patients should receive further stool antigen test, rapid urease test or urea breath test to confirm if there is active infection. Subsequently, patients with concurrent active *H. pylori* infection and sarcopenia should start *H. pylori* eradication therapy with no delay to avoid deterioration of muscle mass.

There are limitations of this study that should be mentioned. First, National Health and Nutrition Examination Survey (NHANES) itself is a cross-sectional study collecting information at a specific point of time, which weakens the causal inference made from this data. In addition, NHANES only collects data from noninstitutionalized US population, and thus the application in other countries or regions need further studies. Second, we utilized blood test results for detecting *H. pylori* infection because other exam methods were not available from NHANES. Other non-invasive tests, urea breath test and stool antigen test, are useful as they can distinguish active infection from past exposure [34]. However, serological tests are proved to be as accurate in untreated patients [35]. As our goal is investigating the relationship between *H. pylori* infection and sarcopenia, we believe adopting both past and current infection of *H. pylori* can still reflect real condition. Third, CagA status are mostly detected by polymerase chain reaction (PCR) in fecal and biopsy samples [36, 37]. Though serological detection is revealed to be sensitive [38], but it is still a relatively indirect mean comparing to PCR.

## Conclusions

Serum *H. pylori* infection markers including anti-*H. pylori* antibody and CagA seropositivity are associated with sarcopenia and low muscle quantity. Once an active *H. pylori* infection is confirmed, patients who are at high risk of sarcopenia may consider receiving *H. pylori* eradication therapy concerning the dual benefit of treating the bacterial infection as well as alleviating the deterioration of muscle mass. Further studies are warranted to verify the application in different races and the possible mechanisms underlying this relationship.

## Methods

### Study population and data collection

Data were obtained from the Third NHANES (NHANES III), which enrolled 39,695 nationwide samples of US population from 1988 to 1994. NHANES is one of the data collection systems of the National Center for Health Statistics (NCHS), a part of the Centers for Disease Control and Prevention (CDC). Different from recent data which is collected and released every 2 years, this 6-year sample was categorized into three released files, containing augmented and surplus data of environmental exposure and sera laboratory components in later years. All study protocols were approved by the Institutional Review Board of CDC, and written informed consent was obtained from each participant. According to our study design, we first included those ≥ 60 years old (n = 5476), as sarcopenia is an age-related disease characterized by decline in muscle mass, strength and function [39]. Then, we excluded those with missing related information of serum *H. pylori* antibody, serum CagA seropositivity, skeletal muscle mass index, and gait speed. After proper selection, 3453 participants were eligible for further analyses.

For determining an adequate sample size to ensure statistical power, we admit a degree of error in the estimate of 5% and a confidence interval of 95%. Based on previous studies, the prevalence of sarcopenia in US population ranges from 45 to 59%. It was calculated as necessary to recruit a minimum number of 371 to 379 subjects to obtain an acceptable estimate (80% power). Thus, in the current study with a total of 3453 participants allows us to make a statistically reliable sample of the prevalence of sarcopenia.

### Definition of sarcopenia and anthropometric measurements

Muscle mass was measured using bioelectrical impedance analysis(BIA) in NHANES III, which is considered an applicable alternative to whole body by dual-energy X-ray absorptiometry (DXA) by international guidelines [40, 41]. The instrument was Valhalla 1990B Bio-Resistance Body Composition Analyzer (Valhalla Scientific, San Diego, CA, USA). We adopted the analytical equation developed by Janssen et al. [42]: skeletal muscle mass (kg) = [(height^2^ (in cm)/ BIA resistance (in ohms, Ω) × 0.401) + (gender × 3.825) + (age (in years) × –0.071)] + 5.102. SMI was further calculated by dividing the skeletal muscle mass above (in kg) by height squared (m^2^). Sarcopenia was defined as having a SMI which is more than one standard deviation (SD) away from sex-specific, healthy population between the ages of 20–39, following similar studies [43, 44]. In our study, the cutoff values of sarcopenia were < 10.76 kg/m^2^ in men and < 6.76 kg/m^2^ in women.

Other components that evaluate impaired physical performance of sarcopenia are as follows. The muscle strength was determined by assessing the ability of the participant to lift or carry 10-pound weight and the degree of muscle strength was graded to no difficulty, some difficulty, much difficulty or unable. Participants answering’ much difficulty’ and ‘unable’ were classified into the low muscle strength group. Gait speed is calculated by the time used for completing 8-feet walk which is carried out in the Mobile Examination Center (MEC).

### Determination of *H. pylori* infection

The status of serum anti-*H. pylori* IgG antibody and serum anti-CagA IgG antibody were obtained from the NHANES III surplus documents. Antibodies to *H. pylori* were assessed using ELISA with Pyloristat kit (Whittaker Bioproducts, Walkersville, Maryland, USA); IgG antibodies to CagA were measured using an optimal ELISA assay using recombinant CagA for the detection of anti-CagA antibody developed by Vanderbilt University [45]. Detailed methodology followed protocols developed by Blaser et al. in 1980s [46].

### Covariates

The details of self-reported demographic information are listed in the following. The BMI was calculated by weight in kilograms divided by height in meters squared (kg/m^2^). Gait speed (m/s) was calculated by dividing the distance of 8-feet walk (2.44 m) by the time (second) used for it. As for serum laboratory results, plasma glucose was measured with a modified hexokinase enzymatic method, and plasma C-reactive protein levels with an automated Behring Nephelometer Analyzer System (Behring Diagnostics, Inc, Somerville, NJ) [47]. The study population were classified based on their race into four categories, non-Hispanic white, non-Hispanic black, Mexican–American, and others (including multi-racial). Positive smoking history was recognized by responding yes to the question” have you ever smoked at least 20 cigarettes in your entire life?” Medical conditions including arthritis, asthma or diabetes mellitus were identified by the fact patients had been diagnosed with or told with the above diseases. All information was collected following standardized protocols formulated on the CDC reference instruments and manuals.

### Study design and statistical analysis

**Fig. 1:**
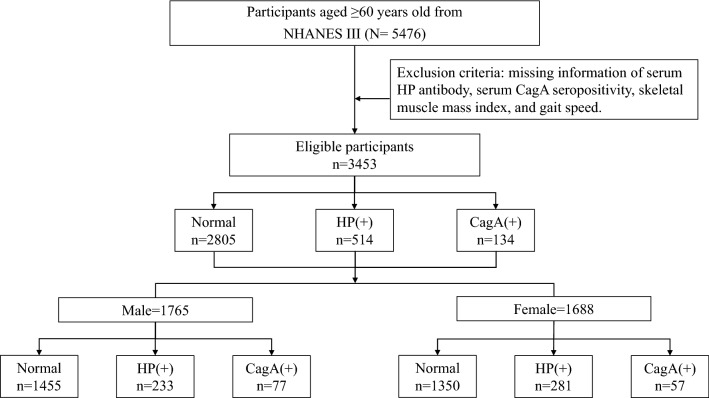
A flow diagram representing patient enrollment and study design of this study

Participants were classified into three groups: normal (without evidence of any *H. pylori* infection), anti-*H. pylori* IgG positive [presented in the context as *H. pylori* (+)], and concurrent anti-*H. pylori* IgG and anti-cytotoxin-associated gene A IgG positive [presented in the context as CagA (+)]. The analytic software used here was SPSS (IBM Corp. Released 2013. IBM SPSS Statistics for Windows, Version 22.0. Armonk, NY: IBM Corp.). Quantitative parameters were indicated as means and SDs, whereas quantitative parameters were indicated as number and percentage (%). Demographic characteristics were compared by an independent t-test; a Student's t-test was used for continuous variables and Chi-square test for categorical variables. Logistic regression was used to predict risk of each subgroup toward qualitative variables such as low gait speed, diagnosis of sarcopenia and having difficulty lifting heavy objects; Linear regression was used to predict risk toward quantitative variables like skeletal muscle index (SMI) and gait speed. Four expanded models were offered for adequate correction, with Model 1 being the unadjusted one, and Model 2,3,4 being adjusted ones with different confounding variables. All p values < 0.05 were considered statistically significant. Sub-analyses of separate gender were also performed. A study flow diagram is presented in Fig. 1.

## Data Availability

The datasets generated and/or analyzed during the current study are publicly available from the NHANES website. (https://wwwn.cdc.gov/nchs/nhanes/nhanes3/default.aspx).

## Abbreviations

*H. pylori*: *Helicobacter pylori*
CagA: Cytotoxin-associated gene A
NHANES III: Third National Health and Nutrition Examination Survey
NCHS: National Center for Health Statistics
CDC: Centers for Disease Control and Prevention
MEC: Mobile Examination Center
BIA: Bioelectrical impedance analysis
DXA: Dual-energy X-ray absorptiometry
SMI: Skeletal muscle index
SD: Standard deviation
BMI: Body mass index
OR: Odds ratio
IL: Interleukin
TNF: Tumor necrosis factor
IF: Interferon
MALT: Mucosa associated lymphoid tissue
PCR: Polymerase chain reaction
